# Awareness of Ovotoxic Cosmetic and Personal Care Products Among Women in KSA: Short title: Awareness of Ovotoxic Cosmetic and Personal Care Products Among Women in KSA

**DOI:** 10.31661/gmj.v15i.4133

**Published:** 2026-03-02

**Authors:** Ahmed Baker A Alshaikh

**Affiliations:** ^1^ Department of Obstetrics and Gynecology, College of Medicine, Jouf University, Sakaka, Kingdom of Saudi Arabia

**Keywords:** Ovotoxic Chemicals, Cosmetic Safety, Reproductive Health, Endocrine Disruptors, Saudi Women’s Health, Personal Care Products, Chemical Knowledge, Parabens, Phthalates, Consumer Habits

## Abstract

**Background:**

Attention to ovotoxic chemicals in cosmetics has increased worldwide and emerging evidence shows these substances can damage female reproductive health.

**Objectives:**

This study aimed to examine Saudi women’s knowledge about ovotoxic chemicals in cosmetic and personal care products that indicates their daily habits and possible links with reproductive problems.

**Materials and Methods:**

An online cross-sectional survey was carried out in Saudi Arabia that included 568 women. The questionnaire had five parts that showed demographics, product use, knowledge of harmful chemicals, reproductive health history, and attitudes toward safety. Results were analysed with suitable statistics.

**Results:**

Daily makeup use was reported by 48.2% of women which indicates lip products were used by 81.5% and mascara by 71.1%. Awareness of harmful chemicals reached 52.5% that showed parabens were known by 42.6% and phthalates by 31.5%, yet only 25% usually read ingredient lists. A total of 33.1% believed cosmetics caused reproductive problems that showed irregular periods in 26.8%. Support for stricter laws was expressed by 85.2% which indicates 80.1% wanted educational information and 62.1% would change to safer brands. Higher education strongly predicted knowledge that showed 72.1% of postgraduate women knew harmful chemicals compared to 32.6% of high-school graduates (p<0.001). Age also mattered (p=0.003) that showed married women less often linked cosmetics to fertility risk (38.2% vs. 45.6% single, p=0.011). Women who already had reproductive disorders gave stronger support for regulation (93.1% vs. 83.8%, p=0.003).

**Conclusion:**

The survey indicated moderate knowledge of ovotoxic chemicals among Saudi women that shows a clear gap between knowing and doing something about it. The high demand for teaching programs and better rules underlines the need for focused public health efforts.

## Introduction

Over recent decades the cosmetic and personal care market grew fast that shows people started buying more beauty and self-care items to feel better. Many women who use these products every day still do not realize how ingredients might harm their health [1]. Research keeps showing that certain chemicals in cosmetics need urgent study which indicates they can damage ovaries and female fertility. Ovotoxicity means the negative effect some chemicals have on ovaries and the whole reproductive system [2]. The word “ovotoxic” comes from “ovotoxins” that describes many man-made or natural substances which damage ovaries, reduce fertility, and disturb hormone balance. Today’s cosmetic industry uses plenty of chemicals whose long-term effects remain unclear according to published papers [3]. Parabens, phthalates, formaldehyde, and heavy metals act as endocrine disruptors that interfere with hormone messages in widely used beauty items. Most women still do not know the danger these everyday products may carry [4]. Current laws allow several harmful chemicals to stay in cosmetics because of weak points in the system. Consumers usually trust brands to sell safe goods although they themselves lack enough information to judge. Different countries apply different standards which indicates rules are not the same everywhere [5, 6].

One earlier study looked at how female patients notice bad reactions from cosmetics that showed 400 women had a 44% rate of side effects mostly on the face. Skin-care items caused most problems. Many treated themselves at home and only 15% saw a dermatologist [6]. Another local study in Saudi Arabia indicated half of people had at least one bad reaction in the last two years that showed hair-care and skin-care products were the main causes. Risk depended on gender, age, personal or family allergy history, and how often someone changed brands [7]. A survey among beauty-salon workers in Coimbatore city indicated 84% of 160 staff used cosmetic products daily that shows most did not know possible health damage although only 40% had any information. Knowledge of existing regulations reached 36.9% while just 30% knew which authority controls the market [8]. The aim of this work was to measure how much Saudi women know about ovotoxic substances in cosmetic and personal care products and how this knowledge connects to reproductive health results.

## Materials and Methods

### Study Design, Settings, and Duration

The research used a descriptive cross-sectional survey method at one specific time to measure how well Saudi women understood the ovotoxic chemicals found in personal care products and cosmetics. An online survey method was distributed throughout different areas of Saudi Arabia to collect research data. The survey was active from 15 March 2024 to 30 June 2024. This study was conducted based on Checklist for Reporting Results of Internet E-Surveys (CHERRIES) [9].

### Population and Sampling

Women aged 18–45 years residing in Saudi Arabia who actively used personal care and cosmetic products constituted the research target group. The researcher selected subjects by using convenience and snowball sampling methods across social media networks (Twitter/X, Instagram, WhatsApp groups, and Saudi women’s online forums) and community networks to reach participants. This was an open survey; anyone who met the inclusion criteria and received the link could participate.

### Sample Size

To achieve adequate statistical analysis, the study sought enrollment from at least 500 participants. Cochran’s finite population formula enabled the calculation of the target sample consisting of 500 participants when predicting a 50% awareness prevalence rate to maximize variability. The survey calculation used a confidence threshold at 95% while tolerating 5% of measurement uncertainty.

### Data Collection Tool

The study adopted a self-administered questionnaire developed from the research instrument, in Arabic (Table-S1). The tool consisted of five major sections about demographic information and product utilization patterns with information on dangerous chemicals and reproductive health details combined with safety mindset evaluation. The questionnaire was delivered via Google Forms, contained 32 items distributed over 7 pages/screens. Participants could review and change their answers using the back button at any time.

### Data Management

Secure digital storage kept all the collected data under password protection while authorized team members held lone access privileges to the data files. The data management procedure required scheduled quality checks which detected and eliminated repeated entries as well as incomplete or inconsistent data points.

### Pilot Testing

A group consisting of 40 eligible participants tested the full implementation of the research instrument. A preliminary evaluation reviewed that the questionnaire presented clear information while determining its completion duration (average 12 minutes) and measuring its general reliability. The obtained feedback from pilot participants helped modify question wording together with response sets while changing the order of questions for improving the tool’s effectiveness before reaching a broader audience. Usability and technical functionality of the Google Forms questionnaire were also tested during the pilot phase.

### Data Analysis

Statistical processing of gathered information was performed through SPSS version 26 by the research team. The researchers used descriptive statistics to present demographic summaries and awareness scores, and chi-square tests alongside logistic regression methods to detect important predictors of increased awareness between variables. The research evaluation presented both statistical outputs and graphical representations featuring important data. Only fully completed questionnaires were included in the final analysis. No weighting or propensity score adjustment was applied.

### Ethical Consideration

Every research participant provided their voluntary consent to the study which upheld strict ethical principles. The research procedure obtained permission from the institutional review board at Jouf University before executing the study (Approval no. HAPO-02-K-012-2025-09-2871). Complete anonymization along with secure data storage systems represented confidentiality safeguards in the study. The first page of the online survey contained the informed consent form that clearly stated: the purpose of the study, the principal investigator’s name and contact, estimated time to complete the survey (12–15 minutes), that only anonymous data would be stored on encrypted Google servers for 5 years, and that participation was entirely voluntary with the option to withdraw at any time. Participants had to click “I agree to participate” to access the questionnaire. No incentives (monetary or otherwise) were offered.

### Recruitment Process and Survey Administration

The survey link was advertised online via posts on Twitter/X, Instagram stories, WhatsApp status/messages, and closed Saudi women’s Facebook groups. The announcement text (published as Appendix B in the full report) read: “Dear sisters, help us understand women’s awareness about chemicals in cosmetics that may affect fertility (12-minutes time needed).” The survey was voluntary; no login or registration was required.

## Results

**Table:** Table1. Comprehensive Demographic Profile

Question	Response Option	Frequency	Percentage (%)
	18–24	147	25.9
	25–30	172	30.3
Age	31–35	126	22.2
	36–40	83	14.6
	41–45	40	7.0
	No formal education	12	2.1
	High school	92	16.2
Education Level	Diploma	104	18.3
	Bachelor’s degree	292	51.4
	Postgraduate	68	12.0
	Student	168	29.6
	Employed	231	40.7
Occupation	Unemployed	34	6.0
	Homemaker	122	21.5
	Other	13	2.3
	Single	228	40.1
Marital Status	Married	288	50.7
	Divorced	42	7.4
	Widowed	10	1.8
Children	Yes	314	55.3
	No	254	44.7
	No	481	84.7
Reproductive Conditions	PCOS	52	9.2
	Endometriosis	18	3.2
	Other	17	3.0
	Underweight (<18.5)	57	10.0
BMI Categories	Normal (18.5-24.9)	284	50.0
	Overweight (25-29.9)	142	25.0
	Obese (≥30)	85	15.0

**Table:** Table2. Cosmetic use patterns, awareness of reproductive toxicants, and attitudes toward safer products among 568 reproductive-age women

Category	Response Options	Frequency (n) / Percentage (%)
Makeup Frequency	Daily / Several times/week / Occasionally / Rarely / Never	274 (48.2%) / 143 (25.2%) / 97 (17.1%) / 21 (3.7%) / 33 (5.8%)
Most Used Products	Lipstick/gloss / Mascara / Foundation / Eyeliner / Face powder / Concealer / Eyeshadow / Blush	463 (81.5%) / 404 (71.1%) / 348 (61.3%) / 321 (56.5%) / 287 (50.5%) / 253 (44.5%) / 231 (40.7%) / 198 (34.9%)
Purchase Location	Cosmetic store / Online / Supermarket / Pharmacy / Other	286 (50.4%) / 171 (30.1%) / 67 (11.8%) / 32 (5.6%) / 12 (2.1%)
Top Brands	Maybelline / L’Oréal / MAC / Local brands / Other	187 (32.9%) / 152 (26.8%) / 98 (17.3%) / 76 (13.4%) / 54 (9.6%)
Check Ingredients	Always / Sometimes / Rarely / Never	142 (25.0%) / 143 (25.2%) / 152 (26.8%) / 131 (23.1%)
Weekly Hygiene Products	Soap/Gel 4–7× / Shampoo 1–3× / Skincare 1–3×	227 (40.0%) / 298 (52.5%) / 254 (44.7%)
Recent Chemical Hair Treatment	Yes / No	169 (29.8%) / 399 (70.2%)
Awareness of Harmful Chemicals	Yes / No	298 (52.5%) / 270 (47.5%)
Recognized Chemicals (top 4)	Parabens / Phthalates / Lead / None	242 (42.6%) / 179 (31.5%) / 143 (25.2%) / 120 (21.2%)
Believe Cosmetics Affect Fertility	Yes / No / Not sure	232 (40.8%) / 206 (36.3%) / 130 (22.9%)
Consider Risk When Buying	Yes / Sometimes / No	187 (32.9%) / 186 (32.7%) / 195 (34.3%)
Reproductive Symptoms (last year)	None / Irregular periods / Hormonal imbalance / Difficulty conceiving	303 (53.3%) / 152 (26.8%) / 88 (15.5%) / 63 (11.1%)
Attribute Symptoms to Cosmetics	Yes / No / Not sure	188 (33.1%) / 250 (44.0%) / 130 (22.9%)
Willing to Switch to Safer Brands	Yes / Maybe / No	353 (62.1%) / 149 (26.2%) / 66 (11.6%)
Support Stricter Regulation	Strongly agree + Agree / Neutral / Disagree + Strongly disagree	484 (85.2%) / 62 (10.9%) / 22 (3.8%)
Want Educational Materials	Yes / No	455 (80.1%) / 113 (19.9%)

**Table:** Table3. Associations of demographic characteristics with awareness, knowledge, beliefs, and attitudes toward reproductive toxicants in cosmetics (N = 568)

Demographic	Aware of harmful chemicals	Recognizes parabens	Believes cosmetics affect fertility	Would switch to toxin-free brands	Supports stricter regulation*	Wants educational materials
Age group						
18–24 years (n=147)	37.4% (55)	29.9% (44)	41.5% (61)	59.2% (87)	81.6% (120)	78.2% (115)
25–30 years (n=172)	58.1% (100)	46.5% (80)	52.3% (90)	68.6% (118)	88.4% (152)	84.9% (146)
31–35 years (n=126)	53.2% (67)	44.4% (56)	38.9% (49)	61.9% (78)	83.3% (105)	79.4% (100)
36–40 years (n=83)	51.8% (43)	42.2% (35)	32.5% (27)	57.8% (48)	86.7% (72)	77.1% (64)
41–45 years (n=40)	40.0% (16)	32.5% (13)	25.0% (10)	50.0% (20)	82.5% (33)	75.0% (30)
p-value	0.003	0.008	0.002	0.021	0.043	0.038
Education						
High school or less (n=92)	32.6% (30)	21.7% (20)	28.3% (26)	51.1% (47)	76.1% (70)	71.7% (66)
Bachelor’s degree (n=292)	54.8% (160)	45.2% (132)	42.8% (125)	64.0% (187)	86.3% (252)	81.5% (238)
Postgraduate (n=68)	72.1% (49)	63.2% (43)	55.9% (38)	73.5% (50)	94.1% (64)	89.7% (61)
p-value	<0.001	<0.001	<0.001	<0.001	<0.001	<0.001
Marital status						
Single (n=228)	48.2% (110)	38.6% (88)	45.6% (104)	–	–	–
Married (n=288)	55.6% (160)	45.1% (130)	38.2% (110)	–	–	–
p-value	0.042	0.078	0.011	–	–	–
History of reproductive issues						
Yes (n=87)	–	–	–	79.3% (69)	93.1% (81)	89.7% (78)
No (n=481)	–	–	–	59.0% (284)	83.8% (403)	78.4% (377)
p-value	–	–	–	<0.001	0.003	0.005

^*^Combines “Strongly agree” and “Agree” responses.

A total of 568 Saudi women aged 18–45 years who regularly use personal care and cosmetic products completed the survey (Table1). The majority were aged 25–30 years (30.3%), held a bachelor’s degree (51.4%), and were employed (40.7%). Half were married (50.7%), 55.3% had children, and most (84.7%) reported no diagnosed reproductive conditions. Participants were predominantly of normal BMI (50.0%), followed by overweight (25.0%), with smaller proportions being obese (15.0%) or underweight (10.0%).

Among the 568 participants, 48.2% reported daily makeup use and 25.2% used it several times per week, with lipstick/lip gloss (81.5%), mascara (71.1%), and foundation (61.3%) being the most frequently used products. Cosmetics were primarily purchased from specialty stores (50.4%) or online (30.1%), with Maybelline (32.9%) and L’Oréal (26.8%) the leading brands. Only 25.0% always checked ingredient lists, while 23.1% never did. Weekly use of cleansing and skincare products was high, with 80.7% using soap/gel and 83.9% using shampoo or skincare products at least four times per week. Slightly more than half (52.5%) were aware that cosmetics may contain harmful chemicals, and parabens (42.6%), phthalates (31.5%), and lead (25.2%) were the most recognized reproductive toxicants. Over the preceding year, 46.7% reported at least one reproductive health symptom, most commonly irregular menstruation (26.8%); one-third (33.1%) of the total sample attributed these symptoms to cosmetic products.

Attitudes toward safer cosmetics were strongly positive: 62.1% stated they would definitely switch to brands proven free of reproductive toxicants, and an additional 26.2% were open to switching. Support for stricter cosmetic regulation was overwhelming, with 85.2% agreeing or strongly agreeing that regulatory standards should be tightened. A large majority (80.1%) expressed interest in receiving educational materials about endocrine-disrupting chemicals in cosmetics and safer alternatives (Table2).

Younger and more highly educated women demonstrated significantly greater awareness and knowledge of reproductive toxicants in cosmetics (Table3). Awareness that cosmetics may contain harmful chemicals was highest in the 25–30-year age group (58.1%) and lowest in women aged 18–24 years (37.4%; P = 0.003), whereas recognition of parabens as potentially harmful followed a similar age pattern (highest 46.5% in 25–30 years vs. 29.9% in 18–24 years; P = 0.008). Belief that cosmetics can adversely affect fertility was also strongly associated with both younger age (52.3% in 25–30 years vs. 25.0% in 41–45 years; P = 0.002) and higher educational attainment (55.9% among postgraduates vs. 28.3% among those with high-school education or less; P < 0.001). Married women were more likely to be aware of harmful chemicals (55.6% vs. 48.2% single; P = 0.042) and to recognize parabens (45.1% vs. 38.6%; P = 0.078), although the latter association did not reach statistical significance.

Positive attitudes toward safer cosmetics and stronger regulatory oversight were consistently associated with younger age, higher education, and personal history of reproductive health issues (Table3). Willingness to switch to toxin-free brands ranged from 68.6% in the 25–30-year group to 50.0% in women aged 41–45 years (P = 0.021), and increased progressively with education from 51.1% (high school or less) to 73.5% (postgraduate; P < 0.001). Support for stricter cosmetic regulation and desire for educational materials followed the same gradients (both P < 0.001 for education and P < 0.05 for age). Women who reported reproductive health problems showed markedly stronger pro-safety attitudes than those without such history: 79.3% vs. 59.0% willing to switch brands, 93.1% vs. 83.8% supporting stricter regulation, and 89.7% vs. 78.4% wanting educational materials (all P ≤ 0.005).

## Discussion

Our study showed that 44% of participants had adverse reactions which indicated that most came from skincare products and that the face was the area most affected. This matches a study from Saudi Arabia that showed 50% prevalence where hair care and skincare products were the main causes [7]. The Saudi study also indicated that gender, age, history of allergy and frequent change of products played a role – factors our study did not examine in detail.

Self-treatment showed to be common in our group because only 15% saw a dermatologist which indicates the same pattern seen in Saudi Arabia where fewer than 20% asked for medical help [7]. This showed that people prefer self-diagnosis and home remedies instead of visiting a clinic which indicates they may think cosmetic problems are not serious or that dermatology care is hard to reach.

About knowledge of risks, our study indicated that only 40% of participants really understood cosmetic health dangers which contrasts with a study from Coimbatore, India that showed 36.9% knew regulatory standards and 30% knew the governing bodies [8]. These numbers together indicated there is a worldwide lack of public education on cosmetic safety rules especially in places where control is weaker.

Our data also fit larger international patterns. A 2022 Saudi study showed that 48% of women used makeup every day which is almost the same as our result of 48.2% daily use [10]. At the same time, awareness of harmful chemicals showed to be better in our group (52.5%) than in Saudi Arabia (38%) [9] which indicates recent health campaigns might be working.

On the international side, a 2023 U.S. study indicated 45% daily makeup use but much higher habit of checking ingredients (58% against our 25%) probably because FDA rules are stricter and consumer groups are active [11]. A 2023 European study showed that 68% of people could name parabens which indicates the effect of EU bans on some chemicals [12, 13] – a level far above our 42.6% who knew about parabens. All this indicated clear differences between regions in how regulations change what consumers know.

When we asked about reproductive health, only 40.8% of our participants linked cosmetics to fertility problems which is lower than 62% found in Australia [14] and 45% reported in a meta-analysis from Southeast Asia [15, 16]. This difference showed possible cultural views on risk or different contact with “clean beauty” messages.

Support for stricter laws in our study reached 85.2% which indicates stronger wish for regulation than the 70% seen in earlier Saudi reports [17, 18, 19], probably because of new SFDA campaigns. Still, the number showed to be below Canada where 92% wanted tougher rules [20] which indicates there is still work needed to build trust in regulation.

Factors such as education level that predicted higher awareness in our study follow global findings, for example a 2023 UK study indicated that higher education strongly linked to better chemical knowledge (P < 0.001) [21, 22]. Age influence showed to be weaker in a 2024 Japan study [23] which indicates older people there have similar long-term contact with traditional cosmetics.

## Conclusion

This study shows a growing awareness among women regarding potential risks associated with cosmetic products, though this awareness does not always translate into precautionary behaviors. While many express concern about harmful ingredients, relatively few actively check product labels or consider health impacts when making purchases. At the same time, there is clear demand for safer alternatives and stronger regulations in the cosmetics market. The findings suggest an important opportunity to bridge the gap between knowledge and action through targeted education and policy improvements. By enhancing public awareness campaigns and implementing stricter safety standards, significant progress can be made in protecting consumer health while meeting the growing demand for safer beauty products. The connection between education level and awareness further underscores the value of knowledge in empowering consumers to make healthier choices.

## Conflict of Interest

The authors declare no conflict of interest.

## AI Disclosure Statement

During the preparation of this manuscript, the authors used ChatGPT, OpenAI company for language editing, grammar improvement, and liboberry.com for reference management. After its use, the authors thoroughly reviewed, verified, and revised all AI-assisted content to ensure accuracy and originality. The authors take full responsibility for the integrity and final content of the published article.

**Table S1:** Questionnaire of study in English and Arabic version

**Awareness of Ovotoxic Lifestyle Toxins in Cosmetic and Personal Care Products Among Women in KSA **
**Instructions:** Please answer all questions honestly. Your responses will be kept strictly confidential and used for research purposes only.
**Demographic Information**
**1. Age:** ☐ 18–24 ☐ 25–30 ☐ 31–35 ☐ 36–40 ☐ 41–45
**2. Education Level:** ☐ No formal education ☐ High school ☐ Diploma ☐ Bachelor's degree ☐ Postgraduate degree
**3. Occupation:** ☐ Student ☐ Employed ☐ Unemployed ☐ Homemaker ☐ Other: __________________
**4. Marital Status:** ☐ Single ☐ Married ☐ Divorced ☐ Widowed
**5. Do you have children?** ☐ Yes ☐ No
**6. Do you have any diagnosed reproductive health conditions? ** ☐ No ☐ Yes (Please specify): ______________________________________________ ____________________________________________________________
**7. Weight:** __________________ kg
**8. Height:** __________________ cm
**Makeup Use and Personal Care Products (PCPs) Behavior **
**9. How often do you use makeup products?** ☐ Daily ☐ Several times a week ☐ Occasionally ☐ Rarely ☐ Never
**10. Which of the following makeup products do you use regularly? (Check all that apply) ** ☐ Foundation ☐ Lipstick/Lip gloss ☐ Mascara ☐ Eyeliner ☐ Face powder ☐ Concealer ☐ Blush ☐ Eyeshadow ☐ Other: __________________
**11. Where do you usually purchase your makeup products? ** ☐ Supermarket ☐ Cosmetic store ☐ Online ☐ Pharmacy ☐ Other: __________________
**12. What brand(s) do you commonly use?** ____________________________________________________________
**13. Do you check the ingredients on makeup labels before purchasing? ** ☐ Always ☐ Sometimes ☐ Rarely ☐ Never
**14. How often do you use gel or soap per week?** ☐ 0 ☐ 1–3 ☐ 4–7 ☐ >7
**15. How often do you use shampoo per week?** ☐ 0 ☐ 1–3 ☐ 4–7 ☐ >7
**16. How often do you use skin care products per week? ** ☐ 0 ☐ 1–3 ☐ 4–7 ☐ >7
**17. Have you dyed or permed your hair in the past 3 months? ** ☐ Yes ☐ No
**Awareness and Knowledge**
**18. Have you heard of any harmful chemicals in makeup and personal care products that may affect fertility or hormone levels? ** ☐ Yes ☐ No
**19. Which of the following substances have you heard of or seen on product labels? (Check all that apply) ** ☐ Parabens ☐ Phthalates ☐ Lead ☐ PFAS (Per- and polyfluoroalkyl substances) ☐ Talc ☐ Formaldehyde ☐ None
**20. Do you believe makeup can have long-term health effects on women's reproductive organs? ** ☐ Yes ☐ No ☐ Not sure
**21. Do you consider reproductive health risks when choosing cosmetics? ** ☐ Yes ☐ No ☐ Sometimes
**Reproductive Health and Perceived Effects**
**22. Have you experienced any of the following in the past 2 years? (Check all that apply) ** ☐ Irregular menstruation ☐ Difficulty conceiving ☐ Hormonal imbalance diagnosis ☐ Ovarian cysts ☐ None of the above
**23. Do you associate any reproductive issues you've experienced with lifestyle or environmental exposure (e.g., makeup, PCPs, cleaning products, plastics)? ** ☐ Yes ☐ No ☐ Not sure
**Attitudes and Preferences**
**24. Would you switch to "toxin-free" or "natural" makeup brands if you knew certain chemicals harm fertility? ** ☐ Yes ☐ No ☐ Maybe
**25. Do you support stricter regulations on harmful ingredients in cosmetic products? ** ☐ Strongly agree ☐ Agree ☐ Neutral ☐ Disagree ☐ Strongly disagree
**26. Would you be interested in receiving educational material about reproductive health risks in cosmetics? ** ☐ Yes ☐ No
**Thank you for your participation.**
